# Physiologically based kinetic modeling of senecionine N-oxide in rats as a new approach methodology to define the effects of dose and endpoint used on relative potency values of pyrrolizidine alkaloid N-oxides

**DOI:** 10.3389/fphar.2023.1125146

**Published:** 2023-03-02

**Authors:** Frances Widjaja, Liang Zheng, Sebastiaan Wesseling, Ivonne M. C. M. Rietjens

**Affiliations:** Division of Toxicology, Wageningen University, Wageningen, Netherlands

**Keywords:** 7-GS-DHP, physiologically based kinetic modeling, pyrrole-protein adducts, rat, relative potency value, senecionine N-oxide, senecionine

## Abstract

Over 1,000 pyrrolizidine alkaloids (PAs) and their N-oxides (PA-N-oxides) occur in 3% of all flowering plants. PA-N-oxides are toxic when reduced to their parent PAs, which are bioactivated into pyrrole intermediates that generate protein- and DNA-adducts resulting in liver toxicity and carcinogenicity. Literature data for senecionine N-oxide in rats indicate that the relative potency (REP) value of this PA-N-oxide compared to its parent PA senecionine varies with the endpoint used. The first endpoint was the ratio between the area under the concentration-time curve (AUC) for senecionine upon dosing senecionine N-oxide or an equimolar dose of senecionine, while the second endpoint was the ratio between the amount for pyrrole-protein adducts formed under these conditions. This study aimed to investigate the mode of action underlying this endpoint dependent REP value for senecionine N-oxide with physiologically based kinetic (PBK) modeling. Results obtained reveal that limitation of 7-GS-DHP adduct formation due to GSH depletion, resulting in increased pyrrole-protein adduct formation, occurs more likely upon high dose oral PA administration than upon an equimolar dose of PA-N-oxide. At high dose levels, this results in a lower REP value when based on pyrrole-protein adduct levels than when based on PA concentrations. At low dose levels, the difference no longer exists. Altogether, the results of the study show how the REP value for senecionine N-oxide depends on dose and endpoint used, and that PBK modeling provides a way to characterize REP values for PA-N-oxides at realistic low dietary exposure levels, thus reducing the need for animal experiments.

## 1 Introduction

Globally, 3% of all flowering plants contain more than 1,000 identified pyrrolizidine alkaloids (PAs) and their N-oxides (PA-N-oxides) (Smith and Culvenor, 1981; Stegelmeier et al., 1999). The toxicity of these PAs originates from their ability to form pyrrole-protein adducts (Ma et al., 2018) and pyrrole-DNA adducts (Xia et al., 2013) upon bioactivation, resulting in liver toxicity such as hepatic sinusoidal obstruction syndrome (HSOS) (Chojkier, 2003; Lin et al., 2011; Gao et al., 2012) and carcinogenicity (Bioassay, 1978; Mattocks and Cabral, 1982; Chan, 2001). PA-N-oxides become toxic upon their reduction to the respective parent PAs by especially microbes of the gastrointestinal tract and by enzymes in the liver (Mattocks, 1971). Yet, although PA-N-oxides are generally considered to be less toxic than their parent PAs, the relative potency (REP) values of PA-N-oxides relative to their corresponding PAs is still under debate.

Previous studies have reported REP values of PA-N-oxides identified based on two approaches. The first approach assumes the REP values of PA-N-oxides to be similar to those of the parent PAs, thus the REP value relative to the parent PA equals 1.0, implying as a worst case approach, that PA-N-oxides are equally toxic to their parent PAs (Merz and Schrenk, 2016). The second approach results in values below 10% of the REP value of the parent PAs, thus the REP values relative to the parent PA are <0.10, where PA-N-oxides are suggested to be substantially less toxic than their parent PAs (Allemang et al., 2018; Louisse et al., 2019; Schrenk et al., 2020). The latter REP values were mainly derived from results of *in vitro* studies that did not take the reduction of PA-N-oxides by intestinal microbiota into account.

REP values derived from *in vivo* data are scarce but do reveal that the REP value for PA-N-oxides relative to PAs may vary with the endpoint used. For example, the data presented in Table 1 for senecionine N-oxide (SENO) and senecionine (SEN) taken from Yang et al. (Yang et al., 2017a) reveal different REP value when calculated using the two different endpoints. The *in vivo* REP value calculated based on the ratio between the area under the concentration-time curve (AUC) for the parent PA upon dosing the PA-N-oxide or an equimolar dose of the PA (Method 1) appears higher (REP value 0.88) than the REP value calculated in a similar way based on the AUC of pyrrole-protein adduct formed (Method 2) (REP value 0.61).

**TABLE 1:** *In vivo* REP values of SENO relative to SEN as calculated by two methods based on different endpoints (see Figure 1 for further details). *In vivo* data for AUC of SEN and AUC of pyrrole-protein adducts formed are extracted from Yang et al. (Yang et al., 2017a).

	Method 1	Method 2
Endpoint used	AUC_SEN_ (min µg mL^-1^)	AUC _pyrrole-protein adducts_ formed (min µg mL^-1^)
Oral SENO dosage 55 μmol kg^-1^ bw	15.11	384.98
Oral SEN dosage 55 μmol kg^-1^ bw	17.24	628.48
REP	0.88	0.61

**FIGURE 1:**
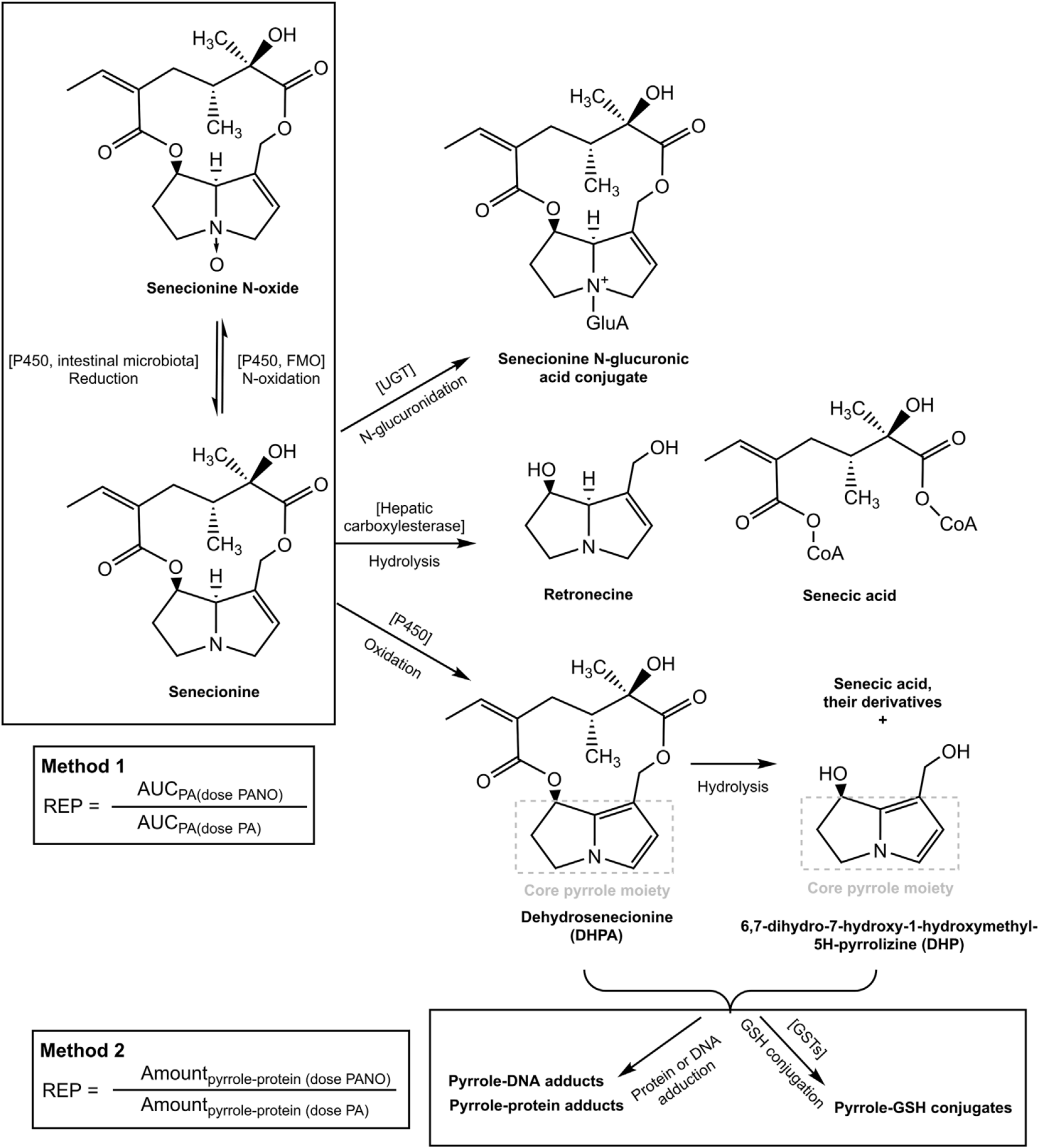
Metabolic pathways for SEN and its N-oxide and the two methods for determining the REP value of the PA-N-oxide relative to the corresponding parent PA. The pathways were adapted from our previous publication (Widjaja et al., 2022a).

Glutathione (GSH)-pyrrole adduct formation might play a role in this discrepancy between the two methods by affecting the formation of pyrrole-DNA/-protein adducts as illustrated in Figure 1. Figure 1 shows that pyrrole-GSH adduct formation, especially formation of 7-GS-DHP as the major adduct (Ning et al., 2019a; Ning et al., 2019b), scavenges the intermediate PA pyrroles (Yang et al., 2016) and a consequential decrease of intracellular GSH levels upon PA exposure has been reported in both *in vivo* and *in vitro* studies. For example, Wang et al. (Wang et al., 2000) administered 160 mg kg^-1^ bw monocrotaline to rats and observed an up to 40% decrease of GSH in rat sinusoidal endothelial cells. Chen et al. (Chen et al., 2009) incubated human normal liver L-02 cells with 100 µM of three PAs, namely, adonifoline, SEN and monocrotaline. Upon treatment with L-buthionine-S-R-sulfoximine (BSO) to deplete intracellular GSH, all three PAs significantly affected cell viability to a further extent than what was observed for the cells not treated with BSO. Recently, Yang et al. (Yang et al., 2016) incubated human hepatic sinusoidal endothelial cells (HSEC) and hepatic parenchymal cells (HepG2 cells), both representing cells that lack cytochromes P450 (CYPs) activity, with 300 µM monocrotaline and its two reactive metabolites, dehydropyrrolizidine alkaloid (DHPA) and dehydroretronecine (DHR). In HSEC, up to 62.5%–75% and 37.5%–75% of the intracellular GSH was depleted after DHPA and DHR exposure, respectively. In contrast to HSEC, only less than 10 and up to 25% GSH was depleted in HepG2 cells after DHPA and DHR exposure. More severe GSH depletion and higher pyrrole-protein adduct levels were observed in HSEC compared to HepG2 cells because HSEC had significantly lower basal GSH level and thus appeared more susceptible towards PA-derived reactive metabolites.

**FIGURE 2:**
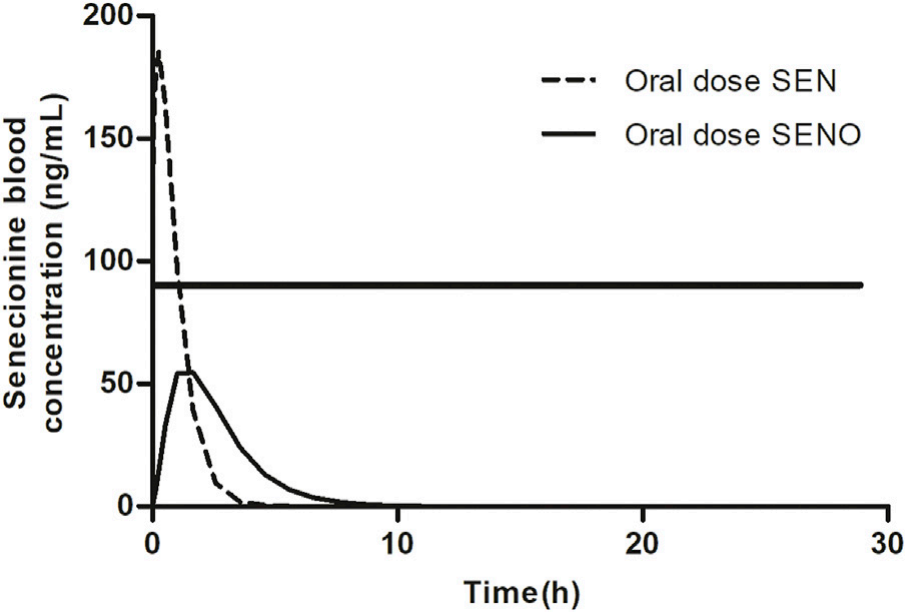
SEN blood concentration *versus* time profile upon dosing SEN or an equimolar concentration of related SENO. A hypothetical threshold (horizontal black line) for the blood SEN concentration indicates when the capacity for GSH based detoxification of the pyrrole intermediate is exceeded. The kinetic profile presented was based on results from our previous publication (Widjaja et al., 2022a).

Based on these observations, it was hypothesized that at high dose levels, high internal levels of PA may result in high levels of reactive pyrrole intermediates resulting in relatively less efficient 7-GS-DHP formation: either because of saturation of the glutathione S-transferase catalyzed GSH conjugation of the pyrrole intermediates, or by depletion of GSH as a result of pyrrole scavenging. As a result, pyrrole-protein adduct formation would become relatively more important. It can be foreseen that this will happen more readily at high internal PA concentrations, and thus that it may occur preferably at high dose of the parent PA and less readily when dosing an equimolar dose of the PA-N-oxide. This is illustrated in Figure 2, where, based on previous *in vivo* and PBK modeling work (Widjaja et al., 2022a; Widjaja et al., 2022b), the PA blood concentration as a function of time is plotted upon dosing an equimolar dose of either the PA-N-oxide or its PA. Assuming the GSH-based pyrrole conjugation capacity would be exceeded above a hypothetical blood PA concentration indicated by the black horizontal line in Figure 2, dosing the PA would yield a substantial period of time where blood concentrations exceed this capacity for GSH pyrrole adduct formation. On the contrary, in this example, the 7-GS-DHP formation capacity would not be exceeded upon an equimolar dose of the PA-N-oxide because the blood concentration of the PA remains below the hypothetical threshold over the entire time period. This can be ascribed to the fact that the PA-N-oxide needs to be reduced to the PA, which takes time resulting in a lower maximum blood concentration (C_max_) of the PA compared to direct PA administration. Saturation or limitation of the 7-GS-DHP formation will result in increased chances for pyrrole-protein adduct formation. If this occurs upon dosing the PA and not upon dosing the PA-N-oxide, the REP value calculated based on the amount of pyrrole-protein adducts formed (Method 2 in Figure 1) will be lower than what will be observed when saturation does not occur of when the REP value is calculated based on the AUC_PA_ (Method 1 in Figure 1).

Overall, the present study aimed to investigate this potential mode of action underlying the different REP values of SENO relative to SEN when calculated by the two methods that use a different endpoint, being either the AUC for the parent PA or the amount of pyrrole-protein adducts. To this end, the GSH conjugation of the reactive pyrrole intermediate was incorporated in the previously developed physiologically based kinetic (PBK) model for SENO and SEN as a new approach methodology (NAM) (Widjaja et al., 2022a). To obtain the required kinetic constants, SEN was incubated with rat liver S9 and GSH to measure the catalytic efficiency (k_cat_) of 7-GS-DHP formation. Subsequently, these *in vitro* data were used as input for the PBK model.

## 2 Materials and methods

### 2.1 Materials

SEN (98%) was purchased from Phytolab (Phytolab GmbH and Co. KG, Germany) and was prepared in DMSO purchased from Acros Organics (Geel, Belgium). Acetonitrile (ACN, UPLC/MS grade) and methanol were purchased from Biosolve (Valkenswaard, the Netherlands). Di-potassium hydrogen phosphate trihydrate (K_2_HPO_4_.3H_2_O) and potassium dihydrogen phosphate (KH_2_PO_4_) were purchased from Merck (Darmstadt, Germany). The reduced form of nicotinamide adenine dinucleotide phosphate (NADPH) was purchased from Carbosynth (Carbosynth, United Kingdom). L-glutathione-reduced (GSH, purity ≥98%) was purchased from Sigma-Aldrich (St. Louis, MO, United States). Pooled rat liver S9 from male Sprague-Dawley (SD) rats was purchased from Corning (Amsterdam, the Netherlands).

### 2.2 *In vitro* rat liver S9 incubations with SEN to form 7-GS-DHP

The rat liver S9 incubation conditions for 7-GS-DHP formation were similar to the conditions previously used to measure SEN depletion (Ning et al., 2019a; Ning et al., 2019b; Widjaja et al., 2022a) but with the addition of GSH. Briefly, the incubation was performed in a total volume of 100 µl containing (final concentrations) 0.1 M potassium phosphate (pH 7.4), 2 mM NADPH, 4 mM GSH, 1 mg/ml rat liver S9, and 0.5–50 µM SEN (added from 50 times concentrated stock solutions in DMSO). Controls were performed without the addition of NADPH. Upon 5 min preincubation with NADPH in a shaking water bath at 37°C, the reaction was started by the addition of SEN. After 60 min incubation, the reaction was terminated by adding 25 µl (20% v/v) ice-cold ACN followed by centrifugation at 16,000 *g* for 5 min at 4°C and supernatants were immediately analyzed by LC-MS/MS.

### 2.3 LC-MS/MS analysis of 7-GS-DHP

7-GS-DHP was quantified by LC-MS/MS using a Shimadzu Nexera XR LC-20AD XR UHPLC system coupled with a Shimadzu LCMS-8040 MS (Kyoto, Japan). A 1 µl aliquot was loaded onto a reverse phase C18 column (Phenomenex 1.7 µm 2.1 × 50 mm). The flow rate was 0.3 ml/min and the mobile phase was made with ultrapure water with 0.1% (v/v) formic acid and ACN containing 0.1% (v/v) formic acid. A linear gradient was applied from 0% to 5% ACN in 8 min and was further increased to 100% ACN in 6 min. This percentage was kept for 0.5 min and was then reduced to the starting conditions in 0.1 min. The column was equilibrated for another 4 min at the starting conditions before the next injection. Under these conditions, 7-GS-DHP eluted at 8.7 min.

For detection, a Shimadzu LCMS-8040 triple quadrupole with an ESI interface was used. The instrument was operated in a positive ionization mode in the multiple reaction monitoring (MRM) mode with a spray voltage of 4.5 kV. The 7-GS-DHP was monitored at the [M + H]^+^ of precursor to products of 443.2 → 425.15 (CE = −7 eV), 443.2 → 118.1 (CE = −24 eV) and 443.2 → 247.2 (CE = −15 eV) *m/z*. The peak area of the total ion chromatogram (TIC) was used for quantification (Ning et al., 2019a; Ning et al., 2019b). Quantification was done by comparing the peak area of the 7-GS-DHP formed in the incubation samples to the calibration curve of 7-GS-DHP ranging from 0.078 to 10 µM (*r* ^*2*^ = 0.991) previously reported by Ning et al. using the same conditions and LC MS-MS instrument (Ning et al., 2019a; Ning et al., 2019b).

### 2.4 Determination of kinetic constants of 7-GS-DHP formation

Kinetic constants were obtained from the SEN concentration dependent rate for 7-GS-DHP formation in incubations with rat liver S9 with SEN concentrations varying from 0.5 to 50 μM, performed as previously reported for lasiocarpine and riddelliine (Ning et al., 2019a; Ning et al., 2019b). The concentration of 7-GS-DHP formed was determined as the concentration detected in full incubations minus the concentration detected in the blanks (without NADPH). Since 7-GS-DHP formation from SEN follows first-order reaction kinetics, the slope of formation rate *versus* substrate concentration directly represents k_cat_, which equals V_max_ and K_m_ values as such, for which can be derived from the Michaelis-Menten equation for substrate concentrations at *[S] << K*_*m*_, thus 1 + *K*_*m*_ */[S]* equals *K*_*m*_ */[S]*. Consequently, the Michaelis-Menten curve in the range where *[S] << K*_*m*_ becomes:

$$v=\frac{V_{\max}}{1+\frac{K_{m}}{\left[S\right]}}=\frac{V_{\max}}{\frac{K_{m}}{\left[S\right]}}=\frac{V_{\max}}{K_{m}}\;\left[S\right]=k_{cat}\;\left[S\right]$$

In this equation *v* is the rate of reaction, *V*_max_ the apparent maximum rate of reaction, *K*_*m*_ the apparent Michaelis-Menten constant, and *[S]* the substrate (SEN) concentration. The slope k_cat_ was determined by fitting the data to the linear regression model using GraphPad (GraphPad Prism software version 5.04, San Diego California United States). The *in vitro* k_cat_ expressed in ml min^-1^ mg^-1^ S9 or the slope value was scaled to an *in vivo* value expressed in L h^-1^ by using an S9 protein yield of 143 mg g^-1^ rat liver, 34 g kg^-1^ bw rat liver and 0.25 kg bw (Punt et al., 2008).

### 2.5 Building the PBK model that includes 7-GS-DHP formation

**FIGURE 3:**
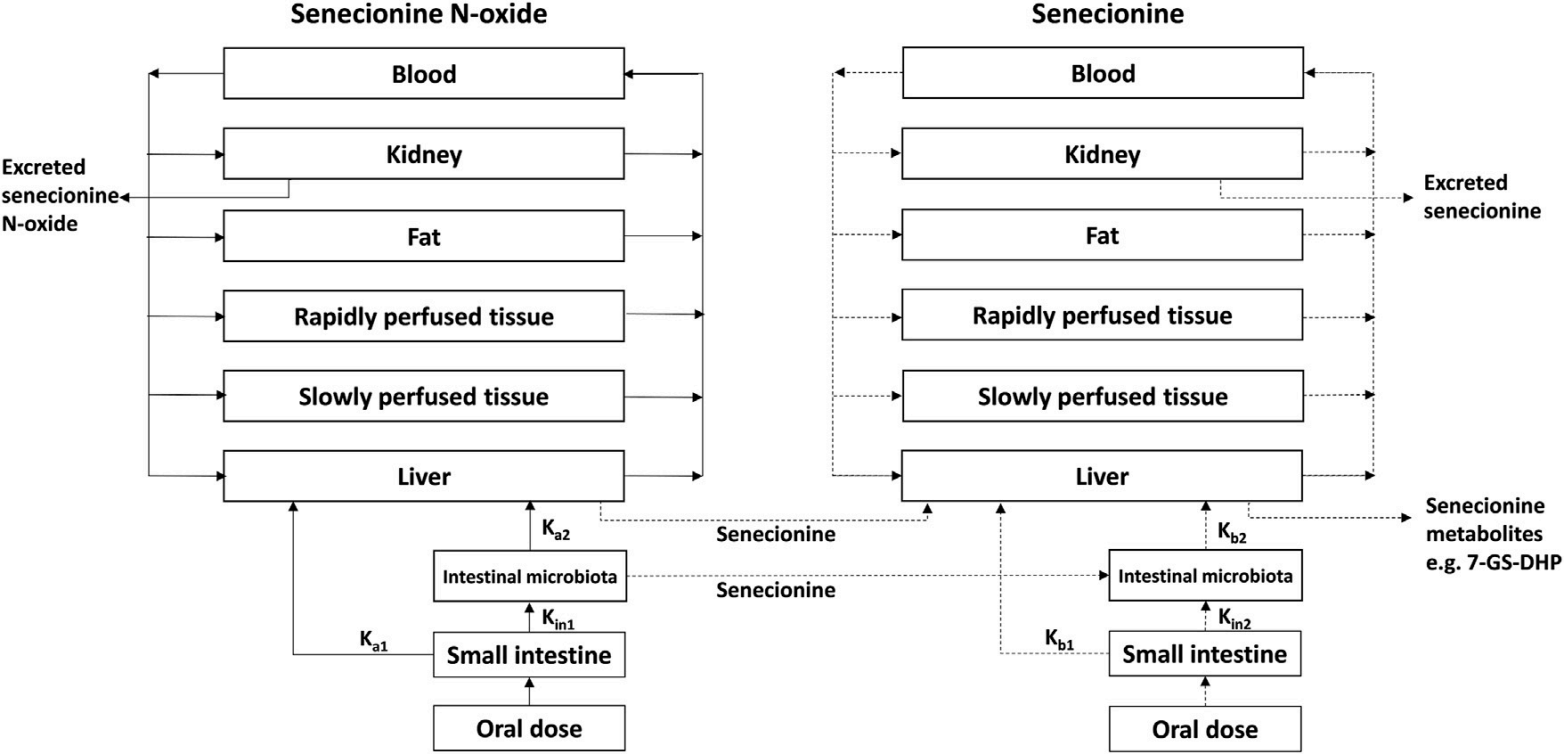
PBK model for SENO with a submodel for SEN as adapted from Widjaja et al. (Widjaja et al., 2022a) with the addition of 7-GS-DHP formation.

The PBK model for SENO with a submodel for SEN in rats previously developed and evaluated (Widjaja et al., 2022a) was built in Berkeley Madonna version 9.1.18 and was run with the Rosenbrock (stiff) method as ordinary differential equations solver. The model was extended to include GSH-scavenging of the reactive pyrrole intermediate through 7-GS-DHP formation from SEN (Ning et al., 2019a; Ning et al., 2019b), with 7-GS-DHP being the major metabolite formed in this reaction (Tamta et al., 2012; Ning et al., 2019a; Ning et al., 2019b) (Figure 3). All other physiological, physicochemical, and kinetic parameters remained unchanged from the previous code. The updated model code can be found in the Supplementary Material. To include 7-GS-DHP formation the equation for the change in the amount of SEN in the liver was extended to read as follows (in bold the parts that were added):

$${AL}_{SEN}^{\prime }=QL*\left({CB}_{SEN}-{CVL}_{SEN}\right)+k_{b1}*{ASI}_{SEN}+k_{b2}*{ALI}_{SEN}+ALM1^{\prime }–\;ALM2^{\prime }–\;ALM4^{\prime }+ALM4^{\prime }$$

where AL_SEN_’ is the change in the total amount of SEN in liver tissue, QL*(CB_SEN_ - CVL_SEN_) is the net amount of SEN going into the liver from SEN that enters from the arterial blood and leaves to the systemic blood circulation, k_b1_*ASI_SEN_ is the amount of SEN entering the liver *via* the portal vein from the small intestine while k_b2_*ALI_SEN_ reflects the uptake of SEN from the large intestine where it is formed from SENO *via* reduction by gut microbiota, ALM1′ is the amount of SEN formed by SENO reduction in the liver, and ALM2′ is the amount of SEN metabolized or cleared in the liver. This part of the equation is similar to what was previously included in the model code.

SEN clearance proceeds by several different pathways including N-oxidation, N-glucuronidation, hydrolysis and oxidation (He et al., 2021). In rat liver S9 incubations containing the cofactor NADPH particularly, N-oxidation, hydrolysis and oxidation are included. Oxidation by cytochromes P450 in these rat liver S9 incubations results in formation of the reactive intermediate DHP and subsequent 7-GS-DHP formation and is thus included in this overall clearance reaction (described by the term -ALM2′). However, to enable quantification of 7-GS-DHP formation the model should also describe this 7-GSH-DPH formation in the liver separately. Therefore, the differential equation that describes the change in the amount of SEN over time should include a term that describes the conversion of SEN into 7-GSH-DPH (the term ALM4′). Given that this conversion described by the term ALM4′, is also included in the overall clearance of SEN (described by the term -ALM2′), one also has to add a term + ALM4′ to the equation to avoid that this part of the clearance is subtracted twice when modeling the change in the amount of SEN in the liver. Thus, subtracting and adding the reaction that forms 7-GS-DHP from the entire SEN clearance (-ALM4′and +ALM4′) allows quantification of 7-GS-DHP formation without disturbing the mass balance of the model. This has previously been published by Ning et al. (2019) (Ning et al., 2019a; Ning et al., 2019b) and the same concept is now applied in the present study.

### 2.6 Calculation of the REP value from the ratio of the AUC for PA or of the amount of pyrrole-protein adducts formed

In the present work, two methods were used to derive the REP value of SENO relative to SEN as presented in Figure 1 and the equations below:

$$Method\;1\;REP=\frac{{AUC}_{SEN\;\left(dose\;SENO\right)}}{{AUC}_{SEN\;\left(dose\;SEN\right)}}$$

$$Method\;2\;REP=\frac{{Amount}_{pyrrole-protein\;adducts\;\left(dose\;SENO\right)}}{{Amount}_{pyrrole-protein\;adducts\;\left(dose\;SEN\right)}}$$

The *in vivo* values for the AUC for SEN and the AUC for the pyrrole-protein adducts required to calculate these two REP values were extracted from Yang et al. (Yang et al., 2017a) (Table 1), while the current PBK modeling predicts the AUC for SEN and the amount for the pyrrole-protein adducts to calculate the REP values. Since the PBK model does not include clearance of the pyrrole-protein adducts, Method 2 uses the total amount instead of the AUC of pyrrole-protein adducts formed. Given that the REP value is calculated as a ratio, the outcome will not be affected regardless of the chosen parameter (as AUC or as amount). Eventually, amount can be converted into AUC and the conversion factor would cancel out when calculating the ratio.

Considering that only a fraction of SEN will be bioactivated to the reactive pyrrole intermediate, and assuming that the bioactivated fraction not binding to DNA is converted to either reactive 7-GS-DHP or pyrrole-protein adducts, the following equations hold:

$${Amount}_{7-GS-DHP\;\left(PA-N-Oxide\right)}+{Amount}_{pyrrole-protein\;adducts\;\left(PA-N-Oxide\right)}=F*\;f*\;{REP}_{Method\;1}*\;{Initial\;dose}_{\left(PA-N-Oxide\right)}$$

$${Amount}_{7-GS-DHP\;\left(PA\right)}+{Amount}_{pyrrole-protein\;adducts\;\left(PA\right)}={F*f*\;Initial\;dose}_{\left(PA\right)}$$

where ${amount}_{7-GS-DHP}$ is the amount of 7-GS-DHP at 24 h (µmol), ${amount}_{pyrrole-protein\;adduct}$ is the amount of pyrrole-protein adducts at 24 h (µmol), *initial dose* is the administrated oral dose level (µmol), *F* is the oral bioavailability amounting to 8.20% (as reported in literature for SEN (Wang et al., 2011) or 100% (for comparison), *f* is the fraction bioactivated to reactive pyrrole intermediates not binding to DNA, *REP*_*Method 1*_ is the REP value of SENO relative to SEN at corresponding dose level based on the ratio of AUC_SEN_ from 0–24 h, PA-N-oxide signifies oral dosing of SENO, and PA signifies oral dosing of SEN. These equations were used to calculate the amount of pyrrole-protein adducts, which were needed to calculate the REP value by Method 2. Upon SENO dosage, SEN will be formed as a result of SENO reduction. Subsequently, formed SEN is bioactivated to its active pyrrole metabolites that give rise to 7-GS-DHP and pyrrole-protein adducts (Figure 1). Similarly, upon SEN dosing, SEN also results in reactive pyrrole metabolites that give rise to 7-GS-DHP and pyrrole-protein adducts. In this approach, pyrrole-DNA adduct formation is considered not to influence the balance between formation of 7-GS-DHP and pyrrole-protein adducts, and to fall outside the bioactivation that is assumed to lead to 7-GS-DHP and pyrrole protein adducts.

### 2.7 Sensitivity analysis

A sensitivity analysis was performed to assess which parameters of the PBK model have the largest impact on the predicted ${amount}_{pyrrole-protein\;adducts}$ , which is the parameter for calculating the Method 2 REP value. A sensitivity analysis for the AUC of SEN, which was the parameter used for calculating the Method 1 REP value, was previously presented (Widjaja et al., 2022a). This previous sensitivity analysis only did not include the k_cat_ for 7-GS-DHP formation. Using the extended model of the present study, the normalized sensitivity coefficients (SC) for this k_cat_ appeared to be 0. Normalized SCs were calculated using the equation below:

$$SC=\frac{\left(C^{\prime }-C\right)/\;C}{\left(P^{\prime }-P\right)/\;P}$$

where *C* is the initial value of the model output, *C′* is the modified value of the model output with a 5% increase of an input parameter, *P* is the initial parameter value, and *P′* is the parameter value with an increase of 5%. Only one parameter was changed each time, while the other parameters were kept at their initial values. A large SC value indicates that the respective parameter has a large impact on the predicted ${amount}_{pyrrole-protein\;adducts}$ . An equimolar dose of 55 μmol kg^-1^ bw (either 19.33 mg kg^-1^ bw SENO or 18.45 mg kg^-1^ bw SEN) reflecting the dose levels used in the animal study of Yang et al. (Yang et al., 2017a) was used to perform the sensitivity analysis in the rat PBK model assuming a bioavailability of 8.20%.

## 3 Results

### 3.1 7-GS-DHP formation from SEN

**FIGURE 4:**
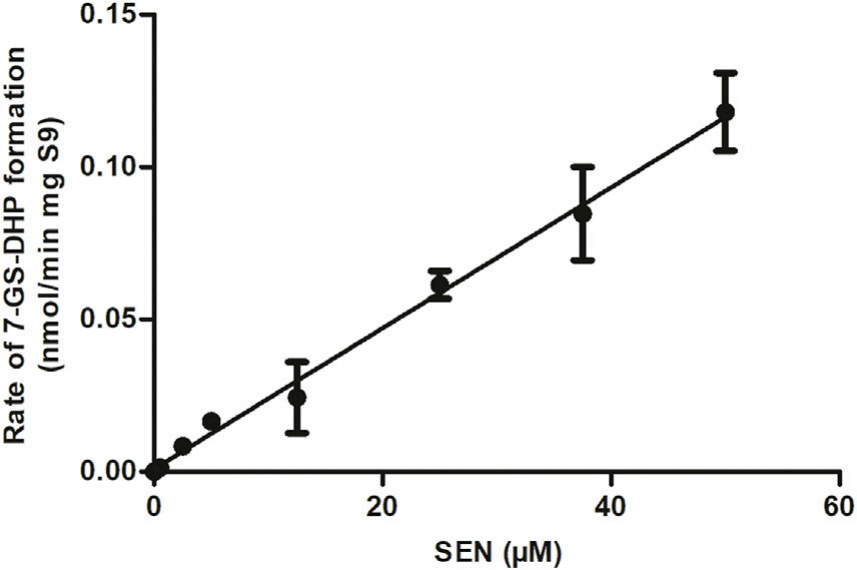
SEN concentration dependent rate of 7-GS-DHP formation in aerobic incubations with rat liver S9. Data are presented as mean ± SD of three independent experiments (*n* = 3).

The rate of formation of 7-GS-DHP in rat liver S9 incubations with increasing concentrations of SEN is shown in Figure 4. The results obtained reveal first-order kinetics with an *in vitro* k_cat_ of 0.0023 mL min^-1^ mg^-1^ S9. When scaled to an *in vivo* value, the k_cat_ amounted to 0.1677 L h^-1^. This *in vitro* k_cat_ value was integrated and converted to an *in vivo* k_cat_ in the PBK model to describe the *in vivo* SEN concentration dependent formation of 7-GS-DHP from either an oral dose of SENO or SEN. Since the initial input parameter was an *in vitro* k_cat_ value, subsequent results will also be presented based on the *in vitro* k_cat_ value which can be converted to the corresponding *in vivo* k_cat_ value using the scaling factor of 72.91 L min h^-1^ mg S9 mL^-1^ that was also implemented in the PBK model. Given that 7-GS-DHP formation is linear (i.e., not saturated) over the SEN concentration range tested up to 50 μM, it is concluded that limitations in 7-GS-DHP formation are unlikely to result from saturation of the kinetics of this conjugation. Limitation of the GSH pyrrole-scavenging capacity may thus rather be attributed to internal GSH depletion as reported to occur in liver cells upon PA or PA-N-oxide exposure (Griffin and Segall, 1987; Neuman et al., 2007; Chen et al., 2009). Therefore, to mimic this GSH depletion and its consequences for 7-GS-DPH adduct formation, the *in vitro* k_cat_ in the SEN submodel was decreased in simulations at high dose levels.

### 3.2 PBK modeling simulation

**FIGURE 5:**
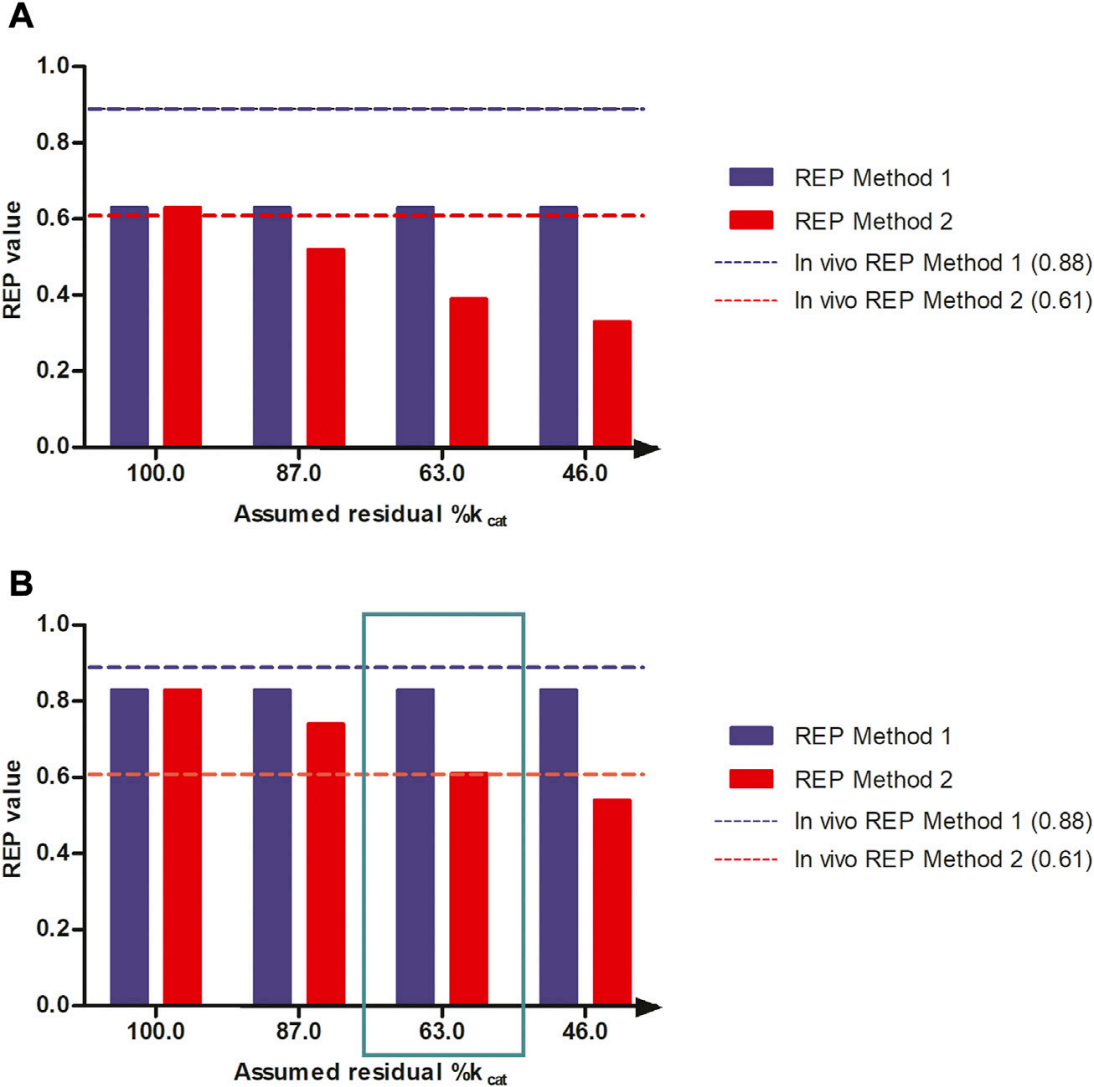
REP values when calculated based on PBK modeling-based predictions with increasing reduction in k_cat_ for 7-GS-DHP formation when dosing SEN because of GSH depletion assuming **(A)** 100% bioavailability and **(B)** 8.20% bioavailability and using either the AUC_PA_ (Method 1, blue bars) or the amount_pyrrole-protein adducts_ (Method 2, red bars). The reduction in k_cat_ mimics the consequences of the GSH depletion for the k_cat_ of pyrrole scavenging by GSH upon dosing SEN. Simulations were performed at a dose level of 55 μmol kg^-1^ bw of SENO and SEN as used by Yang et al. (Yang et al., 2017a) in their *in vivo* rat study. The blue and red horizontal lines represent the REP value derived from the *in vivo* study by Yang et al. (Yang et al., 2017a) (Table 1).

Figure 5 shows the effect of a reduction in the k_cat_ for 7-GS-DHP adduct formation, when dosing the PA, on the calculated REP values at an equimolar dose of 55 μmol kg^-1^ bw of SENO and SEN as used by Yang et al. (Yang et al., 2017a) in their *in vivo* rat study. The REP values were calculated for two bioavailability values, namely, 8.20% bioavailability (Figure 5B) which is in line with literature reported data for the bioavailability of SEN (Wang et al., 2011) and also 100% bioavailability (Figure 5A), the later for comparison. Additional assumptions for the calculations included: a fraction of the dose bioactivated of 0.20, an *in vitro* k_cat_ for 7-GS-DHP formation upon an oral SENO dose of 0.0023 ml min^-1^ mg^-1^ S9, and a value for the *in vitro* k_cat_ for 7-GS-DHP formation upon an oral SEN dose varying from 0.0023 ml min^-1^ mg^-1^ S9 (no GSH depletion and k_cat_ reduction) down to 0.00105 ml min^-1^ mg^-1^ S9 (46% assumed residual k_cat_ due to GSH depletion). At a fraction bioactivated of 0.20 and a k_cat_ for 7-GS-DHP formation of 0.0023 ml min^-1^ mg^-1^ S9, the ratio between 7-GS-DHP and pyrrole-protein adducts formation is 1:1. This implies that it is assumed that the reactive pyrrole intermediate reacts equally well with GSH and protein-SH groups (Mattocks and Jukes, 1992a; Mattocks and Jukes, 1992b). The results obtained reveal that under these conditions, the REP value calculated by Method 2 decreases upon increasing the level of GSH depletion with a concomitant reduction in k_cat_. In addition, with increasing depletion of GSH and thus further reduction of the k_cat_ value for 7-GS-DHP adduct formation upon dosing SEN, the discrepancy between the REP values from Method 1 and 2 becomes more pronounced.

All predictions in Figures 5A,B were compared to the actual REP values derived from the *in vivo* data (Table 1). Figure 5B reveals a similar trend when assuming 8.20% bioavailability and a fraction bioactivated of 0.20. At 8.20% bioavailability, the REP values obtained by Method 1 and 2 are close to the *in vivo* values of 0.88 and 0.61 at 63% residual k_cat_ when dosing SEN, hence a k_cat_ that amounted to 0.00145 mL min^-1^ mg^-1^ S9 (Figure 5B).

**FIGURE 6:**
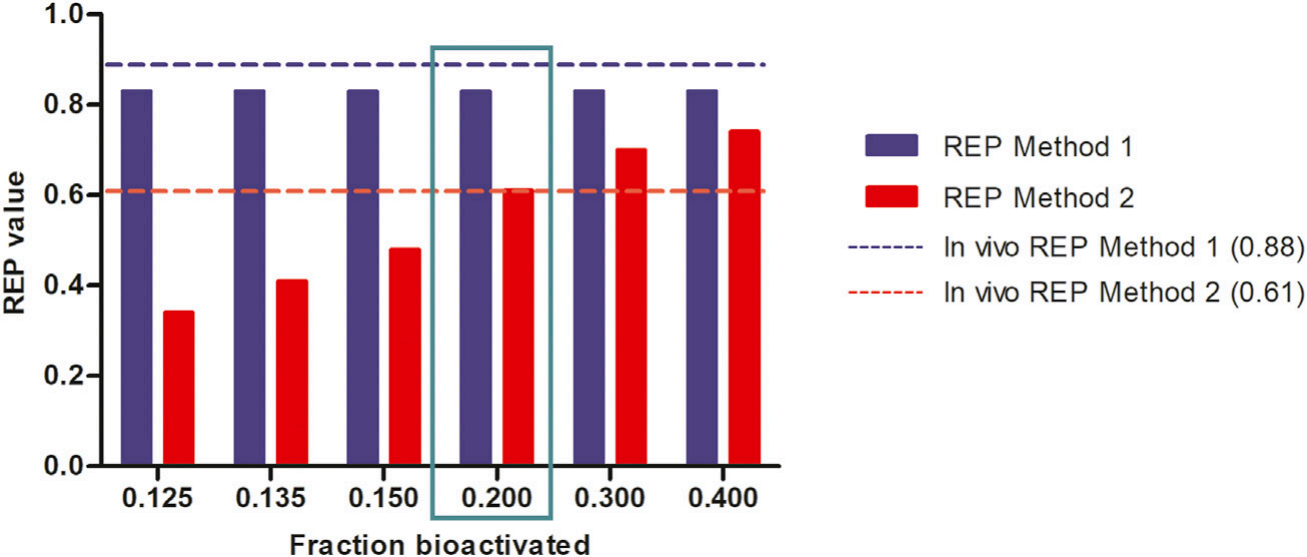
Influence of the assumed fraction bioactivated on REP values when calculated based on PBK modeling-based predictions at a dose level of 55 μmol kg^-1^ bw of SENO and SEN as used by Yang et al. (Yang et al., 2017a) in their *in vivo* rat study assuming 8.20% bioavailability, and using an *in vitro* k_cat_ values of 0.0023 and 0.00145 and mL min^-1^ mg^-1^ S9 for oral SENO and SEN respectively, the latter to reflect GSH depletion upon dosing SEN but not upon dosing SENO.

Figure 6 further shows the PBK-simulated REP values when modifying the calculations with the fraction bioactivated from 0.125 to 0.400 at a dose level of 55 μmol kg^-1^ bw. In this calculation, the amount of 7-GS-DHP was assumed to remain constant at the value obtained with a fraction bioactivated of 0.200 and with a k_cat_ of 0.0023 and 0.00145 ml min^-1^ mg^-1^ S9 for 7-GS-DHP formation when dosing respectively SENO and SEN. As the fraction bioactivated increases, more pyrrole-protein adduct formation occurs when the amount of 7-GS-DHP remains constant. At a low value of fraction bioactivated, the constant level of 7-GS-DHP formation will be higher than the pyrrole-protein adduct formation. In contrast, as the fraction bioactivated increases, more pyrrole-protein adducts are being formed from both oral SENO and SEN dosage. As a result, the REP value increases when the fraction bioactivated increases. Comparison of all the outcomes in Figure 6 reveals that, in agreement with the chosen parameters for Figure 5B, a fraction bioactivated of 0.20 results in REP values that match best with the reported in vivo data.

### 3.3 Dose dependent REP values from method 1 and 2

Next, the consequences for the REP values of modifying the administered dose were studied, in order to obtain insight in REP values not only at the relatively high dose level used in the animal experiment (55 μmol kg^-1^ bw) but also at more realistic low human dietary exposure levels. These simulations were performed selecting the conditions where the REP values at 55 μmol kg^-1^ bw matched the *in vivo* data best, including 8.20% bioavailability, 0.20 fraction bioactivated, and an *in vitro* k_cat_ for an oral SENO and SEN dose of 0.0023 ml min^-1^ mg^-1^ S9 and 0.00145 ml min^-1^ mg^-1^ S9, respectively.

**FIGURE 7:**
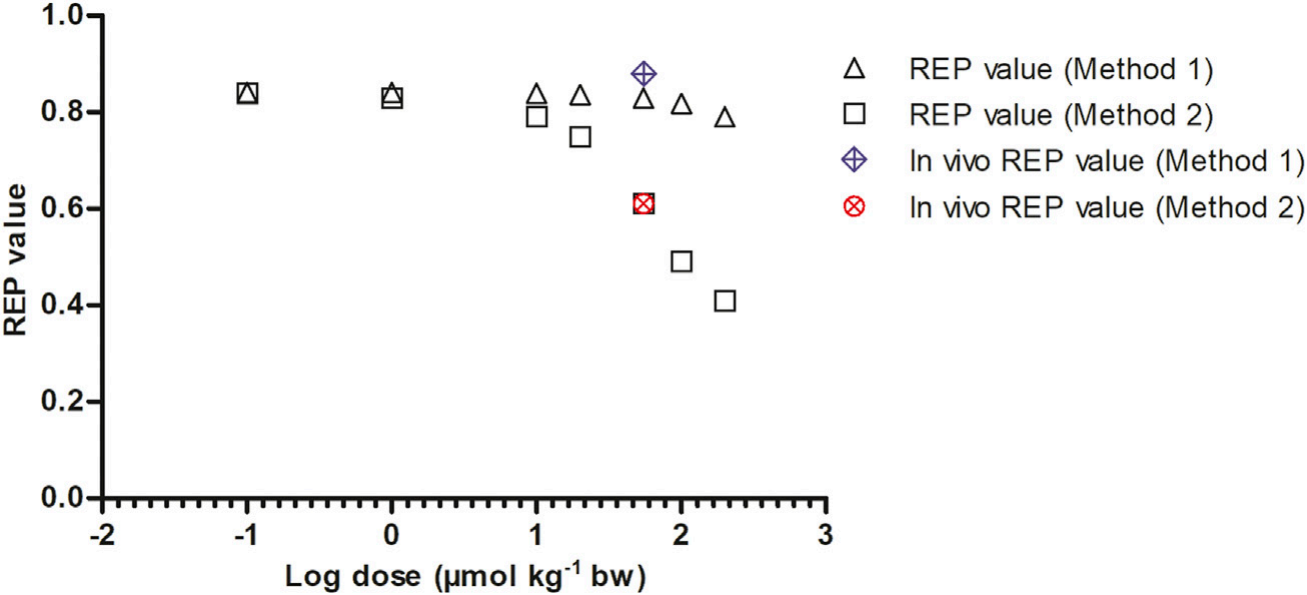
Dose dependent REP values based on Method 1 and 2 as predicted by PBK modeling-based simulations calculated with 8.20% bioavailability and fraction bioactivated of 0.20. *In vivo* REP values were extracted from Yang et al. (Yang et al., 2017a) as explained in Table 1.

To mimic the gradually lower extent of GSH depletion expected upon lowering the dose of SEN, the k_cat_ was linearly increased with reducing dose from 63% assumed residual k_cat_ at 55 μmol kg^-1^ bw to 100% of the original value at 0.1 μmol kg^-1^ bw. In reverse, the k_cat_ was linearly decreased at dose levels above 55 μmol kg^-1^ bw until being 0.01% of the original value at a dose level of 200 μmol kg^-1^ bw. Figure 7 shows the dose-dependent change in the REP values calculated by Method 1 (based on AUC_PA_) and Method 2 (based on amount_pyrrole-protein adducts_). Both Method 1 and 2 show PBK modelling predicted dose-dependent changes in the REP values. At low dose levels, when GSH depletion does no longer play a role, regardless of the chosen method or endpoint, the REP values predicted based on the two endpoints are similar. This is to be expected given that at low dose GSH levels and thus also the k_cat_ for GSH conjugation of the pyrrole adducts is unaffected.

### 3.4 Sensitivity analysis

**FIGURE 8:**
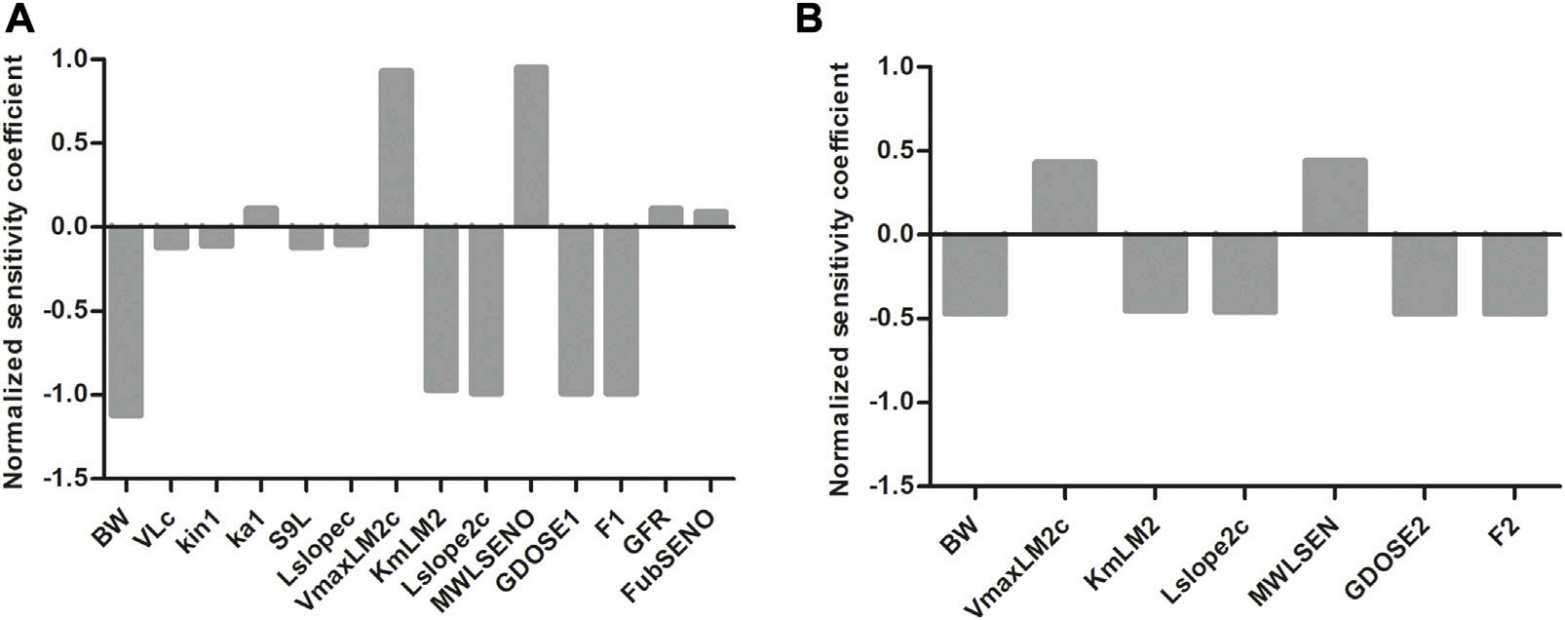
Normalized SCs for the parameters of the rat PBK model for **(A)** SENO and **(B)** SEN dosage on predicted amount_ pyrrole‐protein adducts_ (µmol) at equimolar doses of 55 μmol kg^-1^ bw. The *in vitro* k_cat_ of 7-GS-DHP formation is 0.0023 or 0.00145 mL min^-1^ mg^-1^ S9 for oral SENO or SEN, respectively. The complete list of abbreviations can be found in Table 1S of the Supplementary Material.

To evaluate which parameters are influential to the outcome, normalized SCs were calculated for both the SENO and SEN model at high dose (55 μmol kg^-1^ bw) in rat resulting in the data shown in Figure 8. Similar results were obtained for both models, where the parameters of influence on the model predictions for the ${amount}_{pyrrole-protein\;adduct}$ are those involved in the SEN clearance (VmaxLM2c and KmLM2) and 7-GS-DHP formation (Lslope2c). In the SENO model, parameters other than those related to these two are also shown, albeit with significantly lower influence. It is worth noting that the parameters that are negatively related to 7-GS-DHP are positively related to pyrrole-protein adducts and *vice versa*.

## 4 Discussion

Data from a study on SENO and SEN in rats (Yang et al., 2017a) revealed that the REP value of SENO relative to its corresponding PA varied with the endpoint used to determine the REP value (Table 1). The REP value based on comparison of the AUC of the PA yielded a higher REP value of 0.88 than the REP value of 0.61 obtained based on comparison the AUC of the formed pyrrole-protein adducts. In the present paper, it was investigated to what extent the mechanism behind this observation can be attributed to limitation of the 7-GS-DHP formation capacity upon high internal PA concentrations, a situation shown by previous work to be more likely upon dosing the PA than upon dosing an equimolar dose of the corresponding PA-N-oxide (Widjaja et al., 2022a; Widjaja et al., 2022b) (Figure 2).

Results obtained indicated that up to 50 μM SEN, the 7-GS-DHP formation from SEN showed first-order kinetics with an *in vitro* k_cat_ value amounting to 0.0023 ml min^-1^ mg^-1^ S9 scaled to an *in vivo* k_cat_ value amounting to 0.1677 L h^-1^. Previously, Ning et al. (Ning et al., 2019a; Ning et al., 2019b) measured 7-GS-DHP formation from riddelliine and reported *in vitro* and *in vivo* k_cat_ values that were comparable (1.3-fold higher) to those obtained in present study for SEN. Yet, rather than first-order, the trend reported by Ning et al. showed saturating Michaelis-Menten kinetics. This difference can be attributed to the range of PA concentrations used, which was up to 50 µM SEN in the present study compared to concentrations up to 400 µM riddelliine used by Ning et al. (Ning et al., 2019a). PBK model-based reverse dosimetry using the model of the present study reveals that a 50 μM blood concentration of SEN would originate from a dose level of 452 mg kg^-1^ bw, which is far above the dose levels relevant for the present study. This supports that for the present study, linear kinetics adequately describe the 7-GS-DHP adduct formation from SEN. This result also indicates that at the dose level of 55 μmol kg^-1^ bw used in the rat study of Yang et al. (Yang et al., 2017a), saturation of 7-GS-DHP formation is unlikely to occur. This implies that a dose dependent effect on 7-GS-DHP scavenging may rather originate from depletion of the intracellular GSH levels, resulting in a reduction in the rate of conjugation at high dose levels. This hypothesis was further investigated in the present study by calculating the REP values by Method 1 and 2 at a reduced value of the k_cat_ for 7-GS-DHP adduct formation upon dosing SEN at 55 μmol kg^-1^ bw. The results obtained demonstrate that this assumption can indeed explain the differences in the REP value for SENO relative to its parent SEN at high dose level when based on the ratio between the AUC_PA_ values (Method 1) or on the ratio between the amount of the pyrrole-protein adducts formed (Method 2).

The best fit to the experimental data was obtained assuming the following (Smith and Culvenor, 1981): an oral bioavailability of 8.20% (Stegelmeier et al., 1999), a k_cat_ that remains at 0.0023 ml min^-1^ mg^-1^ S9 upon dosing SENO when no GSH is depleted, and an assumed residual %GSH and resulting residual k_cat_ amounting to 63% of the original values upon dosing SEN at 55 μmol kg^-1^ bw (Ma et al., 2018), a fraction of the PA bioactivated into reactive pyrroles that subsequently bind to either GSH or protein of 0.20, and (Xia et al., 2013) the pyrrole-DNA adduct formation is not affecting the balance between formation of 7-GS-DHP and pyrrole-protein adducts. With respect to these assumptions, the following considerations are of interest. A better fit of the predicted Method 2 REP value and the *in vivo* REP value was obtained when using 8.20% instead of 100% bioavailability. This is consistent with previously predicted REP values in rat by Method 1 that also gave a better fit assuming 8.20% bioavailability (Widjaja et al., 2022a). The fact that a previous study in rats reported this 8.20% as the oral bioavailability of SEN further supports this choice (Wang et al., 2011). Decreasing the k_cat_ to 63% of the original value to mimic GSH depletion is also in agreement with previous publications (Griffin and Segall, 1987; Neuman et al., 2007; Chen et al., 2009) where intracellular GSH levels were reported to amount to 56.52% of control after 72 h of exposure of L-02 cells to 100 µM SEN (Chen et al., 2009), to 55% of control after 1 h of exposure of rat hepatocytes to 480 µM SEN (Griffin and Segall, 1987), or even to 24% of control after 6 h exposure of HepG2 cells to 3 mM of *Senecio latifolius* extracts (Neuman et al., 2007). These studies also justify the use of dose dependent GSH depletion as used in the present study because the GSH depletion upon PA exposure appeared to be concentration dependent (Griffin and Segall, 1987; Nigra and Huxtable, 1992; Wang et al., 2000; Neuman et al., 2007; Ji et al., 2008; Chen et al., 2009; Yang et al., 2016; Yang et al., 2017b; Xiong et al., 2020). Based on 8.20% bioavailability and GSH depletion resulting in 63% residual GSH and corresponding k_cat_, the best-fitting value of the fraction bioactivated appeared to be 0.20. This value is lower than the fraction bioactivated calculated from Yang et al. (Yang et al., 2017a) by dividing the AUC level of pyrrole-protein adducts by the sum of the AUC of SENO, SEN and pyrrole-protein adducts formed, amounting to 0.80. Yet, this latter ratio likely overestimates the fraction bioactivated given that the sum of the AUC of SENO, SEN and pyrrole-protein adducts formed does not represent the total mass balance. Lastly, pyrrole-DNA adduct formation was assumed not to affect the balance between formation of 7-GS-DHP and pyrrole-protein adducts. Xia et al. (Xia et al., 2015) reported that GSH can compete with DNA in forming pyrrole-DNA adducts. However, assuming that pyrrole-DNA adduct formation will compete equally well with GSH and pyrrole-protein adduct formation, pyrrole-DNA adduct formation will not affect the balance between formation of 7-GS-DHP and pyrrole-protein adducts and the corresponding REP values.

Finally, it is important to obtain a REP value for the PA-N-oxides relative to riddelliine as reference compound (REP = 1.0) (Widjaja et al., 2022a). REP values of the PA-N-oxides relative to their parent PAs need to be multiplied by the REP value of the parent PA relative to riddelliine in order to get the full REP value of the PA-N-oxides. This full REP value is required for risk assessment of combined exposure.

In summary, REP values of PA-N-oxides relative to their corresponding parent PAs depend on dose and endpoint used (i.e., AUC_PA_ or amount of pyrrole-protein adducts). At low dose levels, the REP value appears to be independent of the endpoint used. In contrast, at high dose levels as used in animal studies that would enable experimental determination of REP values, the REP values based on formed PAs are higher than the ones based on pyrrole-protein adducts. Results of the present study point at the limited capacity of GSH in scavenging reactive pyrrole intermediates at high dose levels to explain this difference. This limited capacity for 7-GS-DHP formation can best be ascribed not to saturation of the enzyme-catalyzed 7-GS-DHP conjugation, but rather to depletion of intracellular GSH levels especially upon dosing the parent PA. Furthermore the results pointed at a dose dependency of the REP values for both endpoints and reveal that REP values determined in animal experiments at relatively high dose levels may not reflect the situation at relevant human dietary intake levels. All in all, our work demonstrates the strength of using new approach methodology like PBK modeling to replace, reduce and refine the use of animal testing in predicting the REP values of PA-N-oxides in rat based on different endpoints also at low dose levels that are more relevant for human dietary exposure, which cannot easily be determined in animal experiments where high dose levels would be required.

## Data Availability

The original contributions presented in the study are included in the article/Supplementary Material, further inquiries can be directed to the corresponding author.

## Abbreviations

AUC, Area under the concentration-time curve; BSO, L-buthionine-S-R-sulfoximine; C_max_, Maximum blood concentration; CYPs, Cytochromes P450; DHP, 6,7-dihydro-7-hydroxy-1-hydroxymethyl-5H-pyrrolizine; DHR, Dehydroretronecine; DHPA, Dehydropyrrolizidine alkaloid; GSH, Glutathione; HepG2, Hepatic parenchymal; HSEC, Human hepatic sinusoidal endothelial cells; NAM, New approach methodology; PAs, Pyrrolizidine alkaloids; PA-N-oxides, Pyrrolizidine alkaloid N-oxides; PBK, Physiologically based kinetic; REP, Relative potency; SCs, Sensitivity coefficients; SEN, Senecionine; SENO, Senecionine N-oxide; 7-GS-DHP, 7-glutathione-DHP; 3Rs, Replacement, reduction and refinement.
