# Supplementary material for: Physiologically based kinetic modeling of senecionine N-oxide in rats as a new approach methodology to define the effects of dose and endpoint used on relative potency values of pyrrolizidine alkaloid N-oxides
Source: Front Pharmacol. 2023 Mar 2;14:1125146. doi: 10.3389/fphar.2023.1125146 (PMC10017778; doi:10.3389/fphar.2023.1125146)
Supplement: Supplementary file 1 [file Table1.DOCX]

**Physiologically based kinetic modelling predicts similar in vivo relative potency of senecionine N-oxide to senecionine between rat and human at realistic low exposure dose**

*Frances Widjaja, Liang Zheng, Sebastiaan Wesseling, Ivonne MCM Rietjens*

**Division of Toxicology, Wageningen University, PO Box 8000, 6700 EA Wageningen, The Netherlands**

*Corresponding author:

Frances Widjaja

Division of Toxicology, Wageningen University

Stippeneng 4, 6708 WE Wageningen, the Netherlands

Email: [frances1.widjaja@wur.nl](mailto:frances1.widjaja@wur.nl)

**Supplementary Material**

**Table 1S**. List of parameters for PBK model of senecionine N-oxide and of senecionine in rat

| **Parameter** | **Description** | **Unit** | **Value** |
| --- | --- | --- | --- |
| Physiological parameters | | | |
| BW | Body weight of rat | kg | 0.25 |
| VFc | Fraction volume of fat | - | 0.070 |
| VLc | Fraction volume of liver | - | 0.034 |
| VBc | Fraction volume of blood | - | 0.074 |
| VKc | Fraction volume of kidney | - | 0.007 |
| VRc | Fraction volume of rapidly perfused tissue | - | 0.041 |
| VSc | Fraction volume of slowly perfused tissue | - | 0.774 |
| QC | Cardiac output 15* (BW^0.74^) | L h^-1^ | 5.38 |
| QFc | Fraction blood flow to fat | - | 0.070 |
| QLc | Fraction blood flow to liver | - | 0.250 |
| QKc | Fraction blood flow to kidney | - | 0.141 |
| QRc | Fraction blood flow to rapidly perfused tissue (0.76-QLc-QKc) | - | 0.369 |
| QSc | Fraction blood flow to slowly perfused tissue (0.24-QFc) | - | 0.170 |
| Physicochemical parameters | | | |
| LogP_SENO_ | Log P SENO | - | 0.5^a^ |
| PF_SENO_ | Fat/blood partition coefficient SENO | - | 0.27^b^ |
| PL_SENO_ | Liver/blood partition coefficient SENO | - | 0.82^b^ |
| PK_SENO_ | Kidney/blood partition coefficient SENO | - | 0.88^b^ |
| PR_SENO_ | Rapidly perfused tissues/blood partition coefficient SENO | - | 0.91^b^ |
| PS_SENO_ | Slowly perfused tissues/blood partition coefficient SENO | - | 0.72^b^ |
| LogP_SEN_ | Log P SEN | - | 1.1^a^ |
| PF_SEN_ | Fat/blood partition coefficient SEN | - | 0.42^b^ |
| PL_SEN_ | Liver/blood partition coefficient SEN | - | 0.70^b^ |
| PK_SEN_ | Kidney/blood partition coefficient SEN | - | 0.73^b^ |
| PR_SEN_ | Rapidly perfused tissues/ blood partition coefficient SEN | - | 0.79^b^ |
| PS_SEN_ | Slowly perfused tissues/ blood partition coefficient of SEN | - | 0.62^b^ |
| Kinetic parameters | | | |
| kin1 | Transfer rate of SENO from lower ileum to cecum | h^-1^ | 0.46^c^ |
| kin2 | Transfer rate of SEN from lower ileum to cecum | h^-1^ | 0.46^c^ |
| ka1 | Transfer rate of SENO from small intestine to liver | h^-1^ | 0.245^d^ |
| ka2 | Transfer rate of SENO from intestinal microbiota compartment to liver | h^-1^ | 0.245^d^ |
| kb1 | Transfer rate of SEN from small intestine to liver | h^-1^ | 1.552^e^ |
| kb2 | Transfer rate of SEN from intestinal microbiota compartment to liver | h^-1^ | 1.552^e^ |
| FBW | Fraction of feces to bodyweight | g feces (g bw)^-1^ | 0.0164^f^ |
| VmaxLIM1c | Unscaled anaerobic SENO reduction to SEN by intestinal microbiota, Vmax | µmol h^-1^ (g feces)^-1^ | 0.32 |
| KmLIM1 | Anaerobic SENO reduction to SEN by intestinal microbiota, Km | µmol L^-1^ | 35.09 |
| S9L | Liver S9 protein yield | mg S9 (g liver)^-1^ | 143^g^ |
| Lslopec | Unscaled aerobic SENO reduction to SEN by liver S9, Vmax/Km | mL min^-1^ (mg S9)^-1^ | 0.0012 |
| VmaxLM2c | Unscaled aerobic SEN depletion by liver S9, Vmax | nmol min^-1^ (mg S9)^-1^ | 0.56 |
| KmLM2 | Aerobic SEN depletion by liver S9, Km | µmol L^-1^ | 24.39 |
| Lslope2c | Unscaled aerobic 7-GS-DHP formation from SEN by liver S9, Vmax/Km | mL min^-1^ (mg S9)^-1^ | 0.0023 |
| GFR | Glomerular filtration rate 0.0052*BW*60 | L h^-1^ | 0.08^h^ |
| Fub_SENO_ | Fraction unbound of SENO | - | 0.987^i^ |
| Fub_SEN_ | Fraction unbound of SEN | - | 0.491^i^ |
| Run settings | | | |
| MWL_SENO_ | Molecular weight SENO | g mol^-1^ | 351.4 |
| MWL_SEN_ | Molecular weight SEN | g mol^-1^ | 335.4 |
| GDOSE1 | Oral dose SENO | mg (kg bw)^-1^ | 19.33^j^ |
| GDOSE2 | Oral dose SEN | mg (kg bw)^-1^ | 18.45^j^ |
| F1 | Assumed bioavailability of SENO | - | 1 or 0.082 |
| F2 | Assumed bioavailability of SEN | - | 1 or 0.082 |
| Starttime | T-0 or starting time | h | 0 |
| Stoptime | T-final or ending time | h | 24 |

^a^Log Kow or Log P values were extracted from XlogP3 3.0 (Pubchem) in the absence of measured value. Log P were used to calculate partition coefficients and fraction unbound values.

^b^Partition coefficients were calculated using QIVIVE tools developed by Wageningen Food Safety Research (Punt, 2020; Berezhkovskiy, 2004).

^c^Transfer rate for both SENO and SEN from lower ileum to cecum were assumed the same as that of orally administered drugs (Kimura, 2002).

^d^Transfer rate of SENO from small intestine and intestinal microbiota (large intestine) were assumed the same. This value was calculated based on the reported Papp value (Yang, 2020) and Ka of riddelliine (Chen, 2018).

^e^Transfer rate of SEN from small intestine and intestinal microbiota (large intestine) were assumed the same. This value was calculated based on the reported Papp value (Yang, 2020) and Ka of riddelliine (Chen, 2018).

^f^Fraction of feces to bodyweight was extracted from previous literature (Hoskins & Zamcheck, 1968).

^g^Liver S9 fraction was extracted from previous literature (Punt, 2008).

^h^Glomerular filtration rate for rat was calculated as derived from previous literature (Walton, 2003)

^i^Fraction unbound values were calculated using QIVIVE tools developed by Wageningen Food Safety Research (Punt, 2020; Lobell and Sivarajah, 2003)

^j^ Equimolar dose of 19.33 mg/kg bw senecionine N-oxide or 18.45 mg/kg bw senecionine were used as those in in vivo study (Yang, 2017)

;Date : June 2022

;Purpose : PBK model senecionine N-oxide with submodel for senecionine and 7-GS-DHP

;Species : Rat

;Compiled by : Frances Widjaja

;Organisation : Division of Toxicology, Wageningen University and Research, The Netherlands

;================================================================

;Physiological parameters

;================================================================

BW = 0.250 ;body weight rat (kg) (Brown, 1997)

;Tissue fractions (Brown, 1997)

VFc = 0.070 ;fraction of fat tissue

VLc = 0.034 ;fraction of liver

VBc = 0.074 ;fraction of blood

VKc = 0.007 ;fraction of kidney

VRc = 0.041 ;fraction of rapidly perfused tissue

VSc = 0.774 ;fraction of slowly perfused tissue

;total of fractions = 1

;Tissue volumes based on fractions (L or kg)

VF = VFc*BW ;volume of fat tissue

VL = VLc*BW ;volume of liver

VB = VBc*BW ;volume of blood

VK = VKc*BW ;volume of kidney

VR = VRc*BW ;volume of rapidly perfused tissue

VS = VSc*BW ;volume of slowly perfused tissue

;--------------------------------------------------------------------------------------------------------------------

QC = 5.380 ;blood flow rate: cardiac output 15*(BW^0.74) (L/h) (Brown, 1997)

;Fraction of blood flow rate (Brown, 1997)

QFc = 0.070 ;fraction of blood flow to fat tissue

QLc = 0.250 ;fraction of blood flow to liver

QKc = 0.141 ;fraction of blood flow to kidney

QRc = 0.369 ;fraction of blood flow to rapidly perfused tissue (0.76-QLc-QKc)

QSc = 0.170 ;fraction of blood flow to slowly perfused tissue (0.24-QFc)

;total of fractions = 1

;Blood flow rates (L/h)

QF = QFc*QC ;blood flow to fat tissue

QL = QLc*QC ;blood flow to liver

QK = QKc*QC ;blood flow to kidney

QR = QRc*QC ;blood flow to rapidly perfused tissue

QS = QSc*QC ;blood flow to slowly perfused tissue

;================================================================

;Physicochemical Parameters

;================================================================

;Partition coefficients (calculated using QIVIVEtools by WFSR) (Punt, 2020; Berezhkovskiy, 2004)

;Senecionine N-oxide ; Log Kow 0.5 (xLogP3 3.0)

PF_SENO_ = 0.27 ; fat/blood partition coefficient SENO

PL_SENO_ = 0.82 ; liver/ blood partition coefficient SENO

PK_SENO_ = 0.88 ; kidney/blood partition coefficient SENO

PR_SENO_ = 0.91 ; rapidly perfused tissues/ blood partition coefficient SENO

PS_SENO_ = 0.72 ; slowly perfused tissues/ blood partition coefficient SENO

;Senecionine ; Log Kow 1.1 (xLogP3 3.0)

PF_SEN_ = 0.42 ; fat/blood partition coefficient SEN

PL_SEN_ = 0.70 ; liver/ blood partition coefficient SEN

PK_SEN_ = 0.73 ; kidney/blood partition coefficient SEN

PR_SEN_ = 0.79 ; rapidly perfused tissues/ blood partition coefficient SEN

PS_SEN_ = 0.62 ; slowly perfused tissues/ blood partition coefficient of SEN

;================================================================

;Kinetic parameters

;================================================================

;Absorption rates (hr-1)

kin1 = 0.46 ; transfer rate of SENO from lower ileum to cecum (Kimura, 2002)

kin2 = 0.46 ; transfer rate of SEN from lower ileum to cecum (Kimura, 2002)

ka1 = 0.245 ; transfer rate of SENO from small intestine to liver (Yang, 2020; Chen, 2018)

ka2 = 0.245 ; transfer rate of SENO from intestinal microbiota compartment to liver

kb1 = 1.552 ; transfer rate of SEN from small intestine to liver (Yang, 2020; Chen, 2018)

kb2 = 1.552 ; transfer rate of SEN from intestinal microbiota compartment to liver

;To ensure that error1 is 0 and massbalance1 is 1, ensure that kin2 = 0 and kb1=0

;To ensure that error2 is 0 and massbalance2 is 1, ensure that kin1 = 0 and ka1=0

;--------------------------------------------------------------------------------------------------------------------

;Metabolism in intestine

;Large intestine (gut microbiota compartment)

;Scaling factors

FBW = 0.0164 ;fraction of faeces to bodyweight (Hoskins & Zamcheck, 1968)

;Unscaled maximum rate of metabolism (umol hr-1 (g faeces)-1)

VmaxLIM1c = 0.32 ;Vmax for anaerobic SENO reduction to SEN by gut microbiota

;Scaled maximum rate of metabolism (umol hr-1)

VmaxLIM1 = VmaxLIM1c*FBW*BW*1000

;Michaelis Menten constant (umol L-1)

KmLIM1 = 35.09 ;Km for anaerobic SENO reduction to SEN by gut microbiota

;--------------------------------------------------------------------------------------------------------------------

;Metabolism of liver

;Scaling factors

S9L = 143 ;liver S9 fraction (mg S9 protein/g liver) (Punt, 2008)

L = VLc*1000 ;liver (34 g/kg bw)

;Scaled metabolism Vmax/Km of aerobic SENO reduction to SEN (L hr-1)

Lslopec = 0.0012 ;slope of V versus [S] (mL min-1 (mg S9)-1)

Lslope = Lslopec/1000*60*S9L*L*BW;

;Unscaled maximum rate of metabolism (nmol min-1 (mg protein)-1)

VmaxLM2c = 0.56 ;Vmax for SEN substrate depletion in incubations with liver S9

;Scaled maximum rate of metabolism (umol hr-1)

VmaxLM2 = VmaxLM2c/1000*60*S9L*L*BW

;Michaelis Menten constant (umol L-1)

KmLM2 = 24.39 ;Km for SEN substrate depletion in liver S9

;Scaled metabolism Vmax/Km of aerobic SEN conversion to 7-GS-DHP by rat liver S9 (L hr-1)

Lslope2c = 0.0023 ;slope of V versus [S] (mL min-1 (mg S9)-1)

Lslope2 = Lslope2c/1000*60*S9L*L*BW;

;================================================================

;Run settings

;================================================================

;Molecular weight

MWL_SENO_ = 351.4 ;molecular weight senecionine N-oxide

MWL_SEN_ = 335.4 ;molecular weight senecionine

;Given oral dose of either senecionine N-oxide or senecionine

GDOSE1 = 19.33 ;given SENO dose (mg/kg bw)

GDOSE2 = 18.45 ;given SEN dose (mg/kg bw)

ODOSE1 = GDOSE1*1E-3/MWL_SENO_*1E6 ;given SENO dose recalculated (umol/kg bw)

ODOSE2 = GDOSE2*1E-3/MWL_SEN_*1E6 ;given SEN dose recalculated (umol/kg bw)

F1= 1 ;assumed bioavailability (either 1 or 0.082)

F2= 1 ;assumed bioavailability (either 1 or 0.082)

DOSE1 = ODOSE1*BW*F1 ;total SENO dose (umol)

DOSE2 = ODOSE2*BW*F2 ;total SEN dose (umol)

;Time of exposure

Starttime = 0 ;t-0 (hr)

Stoptime = 24 ;t-final (hr)

;================================================================

;Dynamics

;================================================================

;Small intestine compartment

;ASI_SENO_ = amount of SENO remaining in small intestine

ASI_SENO_'= -kin1*ASI_SENO_ - ka1*ASI_SENO_

Init ASI_SENO_ = DOSE1

;--------------------------------------------------------------------------------------------------------------------

;Intestine compartment

;ALI_SENO_ = amount of SENO in large intestine (microbiota compartment) (umol)

ALI_SENO_' = kin1*ASI_SENO_ - ALIM1' - ka2*ALI_SENO_

Init ALI_SENO_ = 0

CLI_SENO_ = ALI_SENO_/(FBW*BW)

;ALIM1 = amount of SENO reduced to SEN in large intestine by gut microbiota (umol)

ALIM1' = VmaxLIM1*CLI_SENO_/(KmLIM1+CLI_SENO_)

Init ALIM1 = 0

;--------------------------------------------------------------------------------------------------------------------

;Liver compartment

;AL_SENO_ = amount of SENO in liver (umol)

AL_SENO_' = QL*(CB_SENO_-CVL_SENO_) - ALM1' + ka1*ASI_SENO_ + ka2*ALI_SENO_

Init AL_SENO_ = 0

CL_SENO_ = AL_SENO_/VL

CVL_SENO_ = CL_SENO_/PL_SENO_

;ALM1 = amount of SENO reduced to SEN in liver in aerobic setting (umol)

ALM1' = Lslope*CVL_SENO_

Init ALM1 = 0

;--------------------------------------------------------------------------------------------------------------------

;Fat compartment

;AF_SENO_ = amount of SENO in fat tissue (umol)

AF_SENO_' = QF*(CB_SENO_-CVF_SENO_)

Init AF_SENO_ = 0

CF_SENO_ = AF_SENO_/VF

CVF_SENO_ = CF_SENO_/PF_SENO_

;--------------------------------------------------------------------------------------------------------------------

;Kidney compartment

;AK_SENO_ = amount of SENO in kidney (umol)

AK_SENO_' = QK*(CB_SENO_-CVK_SENO_) - GF_SENO_'

Init AK_SENO_ = 0

CK_SENO_ = AK_SENO_/VK

CVK_SENO_ = CK_SENO_/PK_SENO_

;GFR = glomerular filtration rate 0.0052*BW*60 (L hr-1) (Walton, 2003)

GFR = 0.08

;GF_SENO_ = glomerular filtration of SENO (umol h-1)

GF_SENO_'=GFR*(CVK_SENO_*Fub_SENO_)

Init GF_SENO_ = 0

;Fub_SENO_ = fraction unbound of SENO ([www.qivivetools.wur.nl](http://www.qivivetools.wur.nl) Lobell and Sivarajah, 2003)

Fub_SENO_ = 0.987

;--------------------------------------------------------------------------------------------------------------------

;Rapidly perfused tissue compartment

;AR_SENO_ = amount of SENO in rapidly perfused tissue (umol)

AR_SENO_' = QR*(CB_SENO_-CVR_SENO_)

Init AR_SENO_ = 0

CR_SENO_ = AR_SENO_/VR

CVR_SENO_ = CR_SENO_/PR_SENO_

;--------------------------------------------------------------------------------------------------------------------

;Slowly perfused tissue compartment

;AS_SENO_ = amount of SENO in slowly perfused tissue (umol)

AS_SENO_' = QS*(CB_SENO_-CVS_SENO_)

Init AS_SENO_ = 0

CS_SENO_ = AS_SENO_/VS

CVS_SENO_ = CS_SENO_/PS_SENO_

;--------------------------------------------------------------------------------------------------------------------

;Blood compartment

;AB_SENO_ = amount of SENO in blood (umol)

AB_SENO_' = QL*CVL_SENO_ + QF*CVF_SENO_ + QK*CVK_SENO_ + QR*CVR_SENO_ + QS*CVS_SENO_ - QC*CB_SENO_

Init AB_SENO_ = 0

CB_SENO_ = AB_SENO_/VB

;AUC_SENO_ = area under the curve of SENO (h umol L-1)

AUC_SENO_' = CB_SENO_

Init AUC_SENO_ = 0

;================================================================

;Sub model compartment of senecionine

;================================================================

;Small intestine compartment

;ASI_SEN_ = amount of SEN remaining in small intestine

ASI_SEN_'= -kin2*ASI_SEN_ - kb1*ASI_SEN_

Init ASI_SEN_ = DOSE2

;--------------------------------------------------------------------------------------------------------------------

;Intestine compartment

;ALI_SEN_ = amount of SEN in large intestine (microbiota compartment) (umol)

ALI_SEN_' = kin2*ASI_SEN_ - kb2*ALI_SEN_ + ALIM1'

Init ALI_SEN_ = 0

CLI_SEN_ = ALI_SEN_/(FBW*BW)

;--------------------------------------------------------------------------------------------------------------------

;Liver compartment

;AL_SEN_ = amount of SEN in liver (umol)

AL_SEN_' = QL*(CB_SEN_ - CVL_SEN_) + kb1*ASI_SEN_ + kb2*ALI_SEN_ + ALM1' - ALM2' - ALM4' + ALM4'

Init AL_SEN_ = 0

CL_SEN_ = AL_SEN_/VL

CVL_SEN_ = CL_SEN_ / PL_SEN_

;ALM2 = amount of SEN metabolized into metabolites in liver (umol)

ALM2' = VmaxLM2*CVL_SEN_/(KmLM2+CVL_SEN_)

Init ALM2 = 0

;ALM4 = amount of 7-GS-DHP from SEN by rat liver S9 (umol)

ALM4' = Lslope2*CVL_SEN_

Init ALM4 = 0

CLM4 = ALM4/VL

CVLM4 = CLM4/PL_SEN_

;--------------------------------------------------------------------------------------------------------------------

;Kidney compartment

;AK_SEN_ = amount of SEN in kidney (umol)

AK_SEN_' = QK*(CB_SEN_-CVK_SEN_) - GF_SEN_'

Init AK_SEN_ = 0

CK_SEN_ = AK_SEN_/VK

CVK_SEN_ = CK_SEN_/PK_SEN_

;GFR = glomerular filtration rate (L hr-1) (Chang 2015)

GFR = 0.08

;GF_SEN_ = glomerular filtration of SEN (umol h-1)

GF_SEN_'=GFR*(CVK_SEN_*Fub_SEN_)

Init GF_SEN_ = 0

;Fub_SEN_ = fraction unbound of SEN ([www.qivivetools.wur.nl](http://www.qivivetools.wur.nl) Lobell and Sivarajah, 2003)

Fub_SEN_ = 0.491

;--------------------------------------------------------------------------------------------------------------------

;Fat compartment

;AF_SEN_ = amount of SEN in fat (umol)

AF_SEN_' = QF*(CB_SEN_-CVF_SEN_)

Init AF_SEN_ = 0

CF_SEN_ = AF_SEN_/VF

CVF_SEN_ = CF_SEN_/PF_SEN_

;--------------------------------------------------------------------------------------------------------------------

;Rapidly perfused tissue compartment

;AR_SEN_ = amount of SEN in rapidly perfused tissue (umol)

AR_SEN_' = QR*(CB_SEN_-CVR_SEN_)

Init AR_SEN_ = 0

CR_SEN_ = AR_SEN_/VR

CVR_SEN_ = CR_SEN_/PR_SEN_

;--------------------------------------------------------------------------------------------------------------------

;Slowly perfused tissue compartment

;AS_SEN_ = amount of SEN in slowly perfused tissue (umol)

AS_SEN_' = QS*(CB_SEN_-CVS_SEN_)

Init AS_SEN_ = 0

CS_SEN_ = AS_SEN_/VS

CVS_SEN_ = CS_SEN_/PS_SEN_

;--------------------------------------------------------------------------------------------------------------------

;Blood compartment

;AB_SEN_ = amount of SEN in blood (umol)

AB_SEN_' = QL*CVL_SEN_ + QK*CVK_SEN_ + QF*CVF_SEN_ + QR*CVR_SEN_ + QS*CVS_SEN_ - QC*CB_SEN_

Init AB_SEN_ = 0

CB_SEN_ = AB_SEN_/VB

;AUC_SEN_ = area under the curve (h umol L-1)

AUC_SEN_' = CB_SEN_

Init AUC_SEN_ = 0

;================================================================

;Mass balance calculations

;================================================================

;SENO oral exposure

Total1=DOSE1

Calculated1 = ASI_SENO_ + ALI_SENO_ + AL_SENO_ + AF_SENO_ + AK_SENO_ + GF_SENO_ + AR_SENO_ + AS_SENO_ + AB_SENO_ + ALI_SEN_ + AL_SEN_ + ALM2 + AF_SEN_ + AK_SEN_ + GF_SEN_ + AR_SEN_ + AS_SEN_ + AB_SEN_

ERROR1 = ((Total1-Calculated1)/Total1 + 1E-30)*100

MASSBBAL1 = Total1-Calculated1 +1

;SEN oral exposure

Total2=DOSE2

Calculated2 = ASI_SEN_ + ALI_SEN_ + AL_SEN_ + ALM2 + AF_SEN_ + AK_SEN_ + GF_SEN_ + AR_SEN_ + AS_SEN_ + AB_SEN_

ERROR2 = ((Total2-Calculated2)/Total2 + 1E-30)*100

MASSBBAL2 = Total2-Calculated2 +1

;================================================================

;Blood concentrations in ng/mL

;================================================================

CB_SENO_ngmL = CB_SENO_*MWL_SENO_

CB_SEN_ngmL = CB_SEN_*MWL_SEN_

AUC_SENO_ngmL = AUC_SENO_*MWL_SENO_

AUC_SEN_ngmL = AUC_SEN_*MWL_SEN_

;================================================================
